# Prognostic Value of Antithrombin Levels in COVID-19 Patients and Impact of Fresh Frozen Plasma Treatment: A Retrospective Study

**DOI:** 10.4274/tjh.galenos.2021.2020.0695

**Published:** 2021-02-25

**Authors:** İlkay Anaklı, Perihan Ergin Özcan, Özlem Polat, Günseli Orhun, Gülçin Hilal Alay, Verda Tuna, Emre Çeliksoy, Mehmet Kılıç, Mutlu Mercan, Achmet Ali, Sevgi Beşışık, Figen Esen

**Affiliations:** 1 İstanbul University, İstanbul Faculty of Medicine, Department of Anesthesiology and Reanimation, İstanbul, Turkey; 2 İstanbul University, İstanbul Faculty of Medicine, Department of Internal Medicine, Division of Hematology, İstanbul, Turkey

**Keywords:** Antithrombin, COVID-19, Fresh frozen plasma, Hypercoagulopathy, Mortality

## Abstract

**Objective:**

The defective interplay between coagulation and inflammation may be the leading cause of intravascular coagulation and organ dysfunction in coronavirus disease-19 (COVID-19) patients. Abnormal coagulation profiles were reported to be associated with poor outcomes. In this study, we assessed the prognostic values of antithrombin (AT) activity levels and the impact of fresh frozen plasma (FFP) treatment on outcome.

**Materials and Methods:**

Conventional coagulation parameters as well as AT activity levels and outcomes of 104 consecutive critically ill acute respiratory distress syndrome (ARDS) patients with laboratory-confirmed COVID-19 disease were retrospectively analyzed. Patients with AT activity below 75% were treated with FFP. Maximum AT activity levels achieved in those patients were recorded.

**Results:**

AT activity levels at admission were significantly lower in nonsurvivors than survivors (73% vs. 81%). The cutoff level for admission AT activity was 79% and 58% was the lowest AT for survival. The outcome in those patients who had AT activity levels above 75% after FFP treatment was better than that of the nonresponding group. As well as AT, admission values of D-dimer, C-reactive protein, and procalcitonin were coagulation and inflammatory parameters among the mortality risk factors.

**Conclusion:**

AT activity could be used as a prognostic marker for survival and organ failure in COVID-19-associated ARDS patients. AT supplementation therapy with FFP in patients with COVID-19-induced hypercoagulopathy may improve thrombosis prophylaxis and thus have an impact on survival.

## Abstract (tr)

**Amaç:**

Koagülasyon ve enflamasyon arasındaki etkileşim, koronavirüs hastalığı-19 (COVID-19) hastalarında damar içi pıhtılaşma ve organ disfonksiyonunun önde gelen nedeni olabilir. Anormal pıhtılaşma profillerinin kötü sonuçlarla ilişkili olduğu bildirilmiştir. Bu çalışmada antitrombin (AT) aktivite düzeylerinin prognostik değerini ve taze donmuş plazma (TDP) tedavisinin klinik sonlanım üzerine etkisini değerlendirdik.

**Gereç ve Yöntemler:**

Laboratuvarda doğrulanmış COVID-19 hastalığı olan 104 kritik akut solunum sıkıntısı sendromu (ARDS) hastasının geleneksel pıhtılaşma parametreleri, AT aktiviteleri ve klinik sonlanımları geriye dönük olarak analiz edildi. AT aktivitesi <%75 olan hastalara TDP uygulandı ve bu hastalarda ulaşılan en yüksek AT aktiviteleri kaydedildi.

**Bulgular:**

Giriş AT aktivitesi seviyeleri, ölenlerde yaşayanlara göre önemli ölçüde düşük kaydedildi (%73 vs. %81). Sağkalım için giriş AT aktivitesi eşik değeri %79, en düşük AT aktivitesi eşik değeri %58 olarak bulundu. TDP uygulamasından sonra AT aktivite düzeyleri %75 ve üzerinde olan hastalardaki klinik sonlanım, <%75 olan hastalardan daha iyiydi. AT’nin yanı sıra giriş D-dimer, C-reaktif protein ve prokalsitonin değerleri, mortalite risk faktörleri arasında yer alan koagülasyon ve enflamasyon parametreleriydi.

**Sonuç:**

AT aktivitesi, COVID-19 ile ilişkili ARDS hastalarında sağkalım ve organ yetmezliği için prognostik belirteç olarak kullanılabilir. COVID-19’un neden olduğu hiperkoagülopatili hastalarda TDP ile antitrombin uygulaması, tromboz profilaksisini iyileştirebilir ve böylelikle sağkalımı olumlu yönde etkileyebilir.

## Introduction

Hypercoagulability related to coronavirus disease-19 (COVID-19) may be associated with acute lung injury, multiple organ failure, and mortality [1,2]. Increased D-dimer concentration is the most typical finding in this coagulopathy mimicking other systemic coagulopathies associated with severe infections [3]. Although laboratory findings appear similar to sepsis-associated disseminated intravascular coagulopathy (DIC), COVID-19 has the distinct feature of being more thrombotic than hemorrhagic [4]. Data from recent postmortem analysis of COVID-19 patients showed endothelial cell activation and microcirculation abnormalities, implicating hypercoagulability in the process of disease aggravation [5]. A substantial proportion of patients develop thrombotic complications due to this uncontrolled immunothrombotic response, which might be a major risk factor related to the development of acute respiratory distress syndrome (ARDS) and progression from ARDS to death.

It is important for clinicians to identify diagnostic and monitoring measures for an effective anticoagulant strategy to manage these patients. Evidence suggests monitoring coagulopathy in patients with COVID-19 by measuring prothrombin time, platelet count, and D-dimer concentrations and using low-molecular-weight heparin (LMWH) for prophylaxis of thromboembolic events (TEs) [6].

Antithrombin (AT) is an endogenous coagulation inhibitor and its levels usually decrease during coagulopathies associated with sepsis and septic shock. There is a body of evidence indicating the prognostic value of AT levels and a large nationwide database study demonstrated that AT administration may be associated with mortality reduction in patients with pneumonia and sepsis-associated DIC [7]. Fresh frozen plasma (FFP) is an effective treatment for AT deficiency whereby it is recommended as a potential source of this factor [8].

Data on AT activity and its treatment during COVID-19-induced coagulopathy is scare. In this retrospective data analysis, we report the prognostic value of AT activity levels in critically ill COVID-19 patients and identify the impact of FFP treatment on TEs and outcome.

## Materials and Methods

Data of 104 ARDS patients (≥18 years old) with laboratory-confirmed COVID-19 infection who were admitted to the four intensive care units of the İstanbul University Hospital from March 19 to June 12, 2020, were included in the analysis. The directive for follow-up of COVID-19 patients was as documented. COVID-19 disease was defined by a positive result on a reverse-transcriptase polymerase chain reaction assay of a nasopharyngeal swab collected by the local hospital health authority. Severe acute respiratory syndrome coronavirus-2 (SARS-CoV-2) pneumonia was diagnosed according to the World Health Organization [9]. ARDS was defined according to the Berlin definitions [10]. Data were collected from available electronic medical records and patient files by the attendees responsible for the research facilities of the Department of Critical Care Medicine of the İstanbul Medical Faculty. The ethics committee of the university hospital approved the study (approval number: 2020/64179) and, due to the nature of the retrospective chart review, waived the need for obtaining written informed consent from individual patients.

We collected demographic, clinical, laboratory, and anticoagulant treatment data of the patients on admission to the ICU. Information recorded included age, sex, admission disease severity [Acute Physiology and Chronic Health Evaluation II (APACHE II) and Sequential Organ Failure Assessment (SOFA) scores], underlying comorbidities (chronic cardiac disease, hypertension, chronic pulmonary disease, diabetes mellitus, chronic renal failure, chronic liver failure, malignancies, cerebrovascular disease, autoimmune disease, and immunosuppressive state), laboratory values [platelet count, ferritin, D-dimer, prothrombin time (PT), activated partial thromboplastin time, AT activity, fibrinogen], and inflammatory markers (C-reactive protein, interleukin 6, procalcitonin). Routine measurements of coagulation parameters as well as AT activity levels were measured once every day during ICU treatment. The normal range of AT activity level in our laboratory is between 75% and 125%. Low AT activity levels (<75%) were treated with a maximum daily dose of 10 mL/kg of FFP (1 mL FFP = 1 IU AT; 1 bag FFP = approximately 250 mL). The lowest AT activity levels before FFP treatment and the highest AT activity levels after FFP treatment as well as the total amount of FFP given were recorded. Besides the AT activity, D-dimer levels were recorded both before and after FFP treatment.

According to the recommendations of the Turkish Ministry of Health, all patients who were admitted to the ICU received 40 mg of LMWH (enoxaparin) twice a day, acetylsalicylic acid (ASA), and dipyridamole for thromboprophylaxis.

### Statistical Analysis

Categorical values were presented as numbers of cases and percentages. They were compared using chi-square tests. The distribution of continuous variables was assessed by kurtosis and skewness, with -1.5 to +1.5 accepted as normal distribution. Normally distributed continuous variables were presented as mean ± standard deviation and compared with independent t-tests. Nonnormally distributed continuous variables were presented as medians and interquartile ranges and compared with the Mann-Whitney U test. Receiver operating characteristic (ROC) curves were generated to determine the ability of AT levels to predict mortality. Values of p<0.05 were considered statistically significant. All statistical analyses were performed using SPSS 15.0 for Windows (SPSS Inc., Chicago, IL, USA).

## Results

The demographic and clinical characteristics of the patients are summarized in Table 1. SOFA and APACHE II scores were higher among nonsurviving patients than survivors (p<0.001). Coagulation and inflammatory markers are depicted in Table 2. Significantly lower levels of admission AT activity were recorded in nonsurvivors compared with survivors (73% vs. 81%; p=0.014). AT levels after FFP treatment were significantly lower in nonsurvivors (76%) than survivors (82%) (p=0.024). There was an increase in AT levels after FFP treatment (from 53% to 80%), and much higher levels were achieved in survivors than nonsurvivors (82% vs. 76%).

The AT activity on admission correlated strongly with neither the APACHE II score (r=-0.137; p=0.20) nor the SOFA score (r=-0.229; p=0.031). Correlations between AT activity and inflammatory markers on ICU admission were analyzed. There was a correlation between AT and D-dimer (r=-0.217; p=0.042).

**Figure 1:**
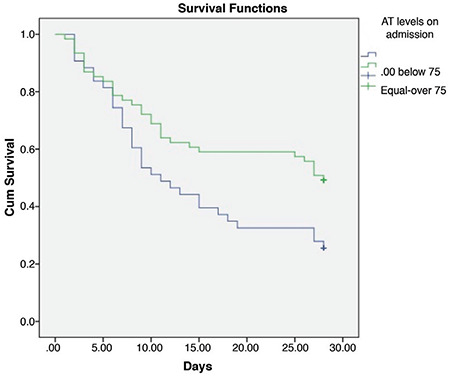
Kaplan-Meier survival curve for overall survival of patients with COVID-19-associated ARDS. Patients whose admission antithrombin (AT) activity levels were below 75% (n=43) and equal to or above 75% (n=61) are represented by blue and green curves, respectively, with shorter survival for patients with AT activity below 75% (p=0.017). ARDS: Acute respiratory distress syndrome, COVID-19: Coronavirus disease-19.

On admission, patients with AT activity of <75% (n=43) had 74% mortality (32/43) and 21% TEs (9/43), while patients with AT activity levels of ≥75% (n=61) had 51% mortality (31/61) and 5% TEs (3/61) (p=0.015 for mortality, p=0.012 for TEs). The Kaplan-Meier plot of survival function over the study period is given for the aforementioned groups in Figure 1. Cumulative survival was higher in patients with AT levels of ≥75% (log-rank chi-square value: 5.67; p=0.017).

**Figure 2:**
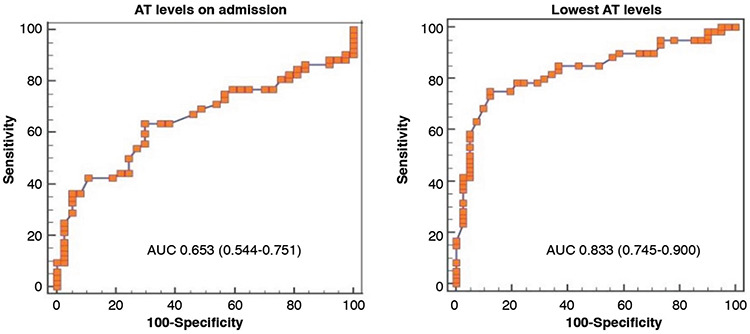
ROC curves for survival predicted by antithrombin (AT) activity levels.

ROC curves were designed according to admission and the lowest AT levels for mortality prediction (Figure 2). The cutoff level for admission AT activity was 79% with 63% sensitivity and 70% specificity. For the lowest AT activity during ICU stay, the cutoff level was 58% with 75% sensitivity and 88% specificity.

There was no significant difference between survivors and nonsurvivors in terms of anticoagulant therapy. Twelve patients (12%) experienced TEs including acute coronary syndrome (n=2), acute limb ischemia (n=4), deep vein thrombosis (n=1), extracorporeal membrane oxygenation cannula clotting (n=2), ischemic hepatitis (n=1), thalamus ischemia (n=1), and cardiac thrombus (n=1) associated with COVID-19. Eleven of those patients (92%) died. The mortality of patients with TEs was significantly higher than that of the patients without TEs (p=0.019). The median admission AT activity for these patients was 67% (38%-87%) and the lowest AT activity was 49% (29%-72%). The median admission AT activity for non-TE patients was 79% (25%-119%) and the lowest AT activity level was 57% (16%-106%) for these patients.

Forty of 43 patients who had AT activity of <75% were treated with FFP; three were excluded because of death before the beginning of FFP treatment. During the ICU stay, the median FFP dose was 6 (3-12) bags. After FFP treatment, AT activity increased on average by 32% from baseline. Those patients were grouped as having AT levels of ≥75% or <75% after FFP treatment (Table 3). There was no significant difference between groups for mortality but TEs were not seen in those patients who had AT activity of ≥75% after FFP treatment.

 D-Dimer levels were recorded at the beginning of FFP treatment and after FFP treatment. At the beginning of FFP treatment, the median D-dimer level was 2624 µg/L (1153-4645 µg/L). After FFP treatment, the lowest median D-dimer level was 1860 µg/L (1085-2908 µg/L) and there was a significant decrease in D-dimer levels (p<0.001).

## Discussion

The main finding of this retrospective analysis is that low levels of AT activity together with other coagulation parameters are associated with mortality in COVID-19-associated ARDS patients requiring intensive care treatment. This finding was in accordance with previous reports suggesting lower levels of AT activity in nonsurvivors. However, reported plasma concentrations rarely dropped below 80% of normal in those studies (84% in nonsurvivors vs. 91% in survivors) [1]. The values of AT in the different severity groups of patients indicated lower levels in critically ill COVID-19 patients; however, the difference was not significant [11]. Our ICU admission AT levels were much lower than these results (73% in nonsurvivors vs. 81% in survivors). This difference is most probably due to the timeframe and the disease severity of the patients included.

Coagulation dysfunction was reported to be a major risk factor for the development of ARDS and hypercoagulation associated with COVID-19 may lead to ARDS and death in patients [3]. Similarly, our nonsurviving ARDS patients had significantly higher D-dimer (2720 µg/L vs. 1370 µg/L) and lower AT levels (73% vs. 81%) compared with the surviving ARDS patients, where the lowest AT levels dropped as low as 43% during the ICU treatment period.

High levels of D-dimer and fibrinogen have been previously discussed as signs of a hypercoagulable state in COVID-19 disease [1,4,12]. Low AT levels, however, have not been previously addressed, which has caused patients to be resistant to anticoagulant therapy and made prophylactic LMWH doses likely inadequate in some patients.

Few reports suggested a possible use of AT supplementation given the low levels of AT in COVID-19 patients. The importance of AT monitoring was emphasized in a cohort of 10 critically ill COVID-19 patients requiring mechanical ventilation. Reduced AT levels resulted in thrombosis despite prophylactic doses of LMWH, suggesting that AT deficiency can be severe [13]. In another study, the median level of AT was significantly lower in the venous thromboembolism group when compared with the non-venous thromboembolism group. However, median levels were within the normal range for both groups [14]. In a study including 16 patients with COVID-19-associated pneumonia and ARDS, reduced AT levels were reported on admission, where four patients had AT levels below the lower limit of the normal range. AT levels then significantly increased, with two patients receiving AT concentrate supplementation that resulted in a significant decrease in fibrinogen levels and D-dimers and of the viscoelastic parameters related to clot strength [4].

Low AT levels were associated with poor prognosis and development of DIC in sepsis and multiorgan dysfunction. Several recent studies revealed that low-dose AT supplementation therapy (1500 IU/day for 3 days) improved treatment outcomes in patients with sepsis-induced DIC and decreased AT activity [15]. In Japan, AT supplementation therapy has been used for patients with sepsis-induced DIC whose AT levels were <70%, but there is no clear threshold for optimal AT activity to start therapy [16]. However, in a multicenter retrospective observational study of patients with sepsis-induced DIC, very low AT activity (43%) was reported as optimal for selecting AT supplementation therapy candidates [7].

Historically, treatment of heparin resistance (HR) associated with low AT has included administration of FFP [17]. Administration of an AT concentrate has reemerged as an alternative treatment of HR and is widely used for AT substitution in cases of severe sepsis [18]. AT concentrates could also be an effective supportive strategy for the management of patients with severe COVID-19 [19]. We treated our COVID-19 patients with FFP when routine daily measurement of AT activity was <75%. We calculated 1 mL of FFP as 1 IU of AT concentrate and 1 bag of FFP as approximately 250 mL. We had no choice other than FFP since we do not have the available AT concentrates in our country. Knowing the risks of FFP (transfusion-related acute lung injury, transfusion-related infections, and volume load), we kept our treatment limits to not more than 750 mL per day of substitution. We observed an increase in AT levels after FFP treatment (from 53% to 80%), and much higher levels were achieved in survivors than nonsurvivors (82% vs. 76%).

In addition to its anticoagulant effect, AT also has strong antiinflammatory properties that are independent of its anticoagulant characteristics and may play a central role in mediating inflammation [20]. In their studies into the intracellular mechanisms that underlie the antiinflammatory effects of AT, Oelschlager et al. [21] confirmed that AT inhibits activation of nuclear factor-κB in cultured human monocytes and endothelial cells [22]. This mechanism represents a plausible explanation for the antiinflammatory effects described. Our results did not show any strong correlation between AT activity levels and inflammatory markers indicating any antiinflammatory impact on the severity of a cytokine storm. However, a significant association of AT levels was found with D-dimer levels, suggesting that AT also has a similar share in reflecting hypercoagulopathy in the COVID-19 clinical setting. We observed decreases in D-dimer levels after FFP treatment. This was in accordance with previous reports, including improved DIC scores in sepsis patients treated with AT [23].

Considerably high incidence of TEs associated with COVID-19 was reported despite prophylactic or therapeutic anticoagulation. A multicenter study of 150 COVID-19-associated ARDS patients demonstrated a 43% prevalence of thrombosis, where 70% of the patients were receiving therapeutic doses of heparin [24]. Our TE ratio was lower than those of previous studies, most probably due to our anticoagulation strategy including ASA (86% of patients) and dipyridamole (76% of patients) in addition to prophylactic heparin.

### Study Limitations

This study has certain limitations. First, the study was conducted retrospectively in an observational manner. Second, we do not have the viral serum levels in order to evaluate viremia, so we are not able to correlate the hypercoagulopathy and outcome with viral load. Third, secondary infection data are not included in the study. Sepsis-induced coagulopathies might have had an additional influence on the coagulation profile in those patients with other sites of infection. Lastly, we did not have a standardized assessment of TEs, which might have resulted in underestimation of the events due to the treating physician’s clinical decision.

## Conclusion

Acute AT deficiency can be severe and may contribute to both the development of thrombosis and failure to achieve maintained therapeutic anticoagulation in patients with COVID-19. AT supplementation may increase the anticoagulant effect of unfractionated heparin or LMWH without increasing heparin dosages. However, the elevation of AT levels to normal values in our patients would require large and repeated doses of AT concentrate, incurring significant cost. FFP treatment can be an alternative like in our case in those countries where AT is not available or if resource allocation is constrained.

Based on the present data, AT levels could be used as a prognostic marker for survival in critically ill COVID-19-associated ARDS patients and we conclude that AT activity levels could be beneficial to consider along with routine coagulation panels. Achieving normal levels of AT may prevent thrombotic events and may improve survival in COVID-19 patients.

## Figures and Tables

**Table 1:**
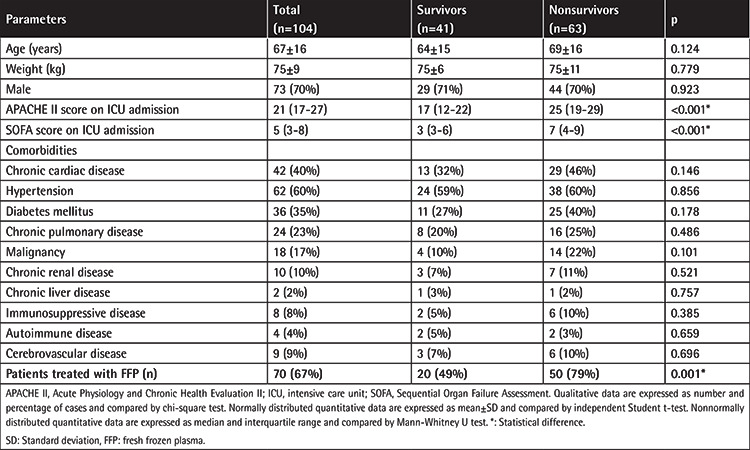
Demographic and clinical characteristics of the patients.

**Table 2:**
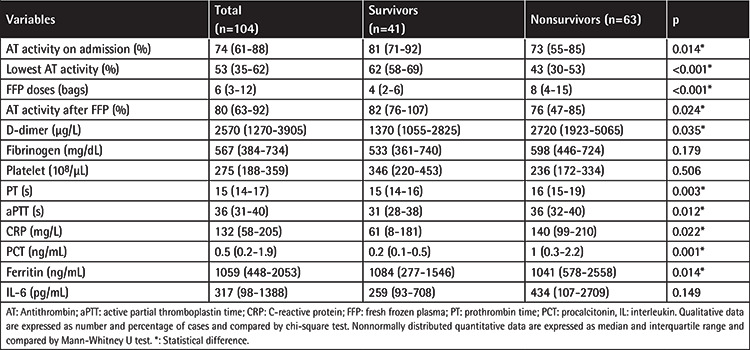
Laboratory parameters and FFP treatment.

**Table 3:**
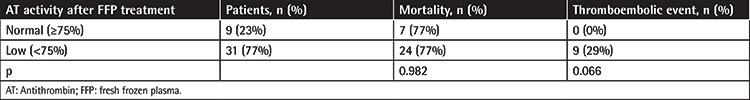
Mortality and thromboembolic events according to AT activity levels after FFP treatment.
